# The Twists and Turns of Diagnosis and Treatment of Pediatric Neuro-Behcet's Disease: A Case Report and Literature Review

**DOI:** 10.3389/fped.2021.769096

**Published:** 2021-12-15

**Authors:** Qiao Zhang, Yizhen Luo, Jianli Zhou, Shaoming Zhou, Zhaoxia Wang

**Affiliations:** ^1^Division of Gastroenterology, Shenzhen Children's Hospital, Shenzhen, China; ^2^Department of Radiology, Shenzhen Children's Hospital, Shenzhen, China

**Keywords:** child, cerebral venous sinus thrombosis, Neuro-Behcet's disease, diagnosis, Crohn disease

## Abstract

**Background:** The neurological manifestation of Behcet's disease (BD) is known as Neuro-Behcet's disease (NBD). The lack of a specific diagnostic method for NBD renders the diagnosis and treatment of NBD challenging.

**Methods and Results:** We report a boy aged 11 years and 11 months who underwent right-eye intraocular lens implantation, appendectomy, perianal abscess removal, thalidomide therapy, and infliximab infusions for his Crohn disease. Magnetic resonance venography (MRV) and magnetic resonance imaging (MRI) were performed to address the onset of headache during the course of his treatment, and cerebral venous sinus thrombosis was detected. After the diagnosis of NBD, the patient was treated with anticoagulation therapy (nadroparin calcium), low-dose corticosteroids, and an immunosuppressant (cyclophosphamide), and consequently, he recovered.

**Conclusion:** This case report shows that NBD is prone to misdiagnosis and missed diagnosis and should be diagnosed based on clinical manifestations and results from colonoscopy, pathological examination, and MRI or MRV.

## Introduction

Behcet's disease (BD) was first reported in 1937 by the Turkish dermatologist Hulusi Behcet. In 1941, Knapp wrote the first clinical case of neurological manifestation of BD, which was later termed Neuro-Behcet's disease (NBD) by Cavara and D'Ermo (1). BD becomes NBD upon the neurological involvement (2). NBD is relatively rare in children and difficult to diagnose, causing delayed diagnosis and treatment.

BD is a systemic inflammatory disease with clinical characteristics of autoinflammatory diseases and vasculitis (3). The main clinical features include recurrent oral ulcers, genital ulcers, and uveitis, and they can affect multiple parts of the body, such as the skin, joints, and cardiovascular, digestive, and nervous systems (4). Previous studies have shown that this condition is related to human leukocyte antigen-B51 (HLA-B51) (5). Cerebral venous sinus thrombosis (CVST) is the most common manifestation of NBD in children (6).

Maldini et al. (7) have estimated that the global prevalence of BD in adults is approximately 10.3 per 100,000 people, with different prevalence rates in different countries. The incidence rate of BD in children is unclear. Nevertheless, 4–26 % of BD cases have been reported to occur before the age of 16 years (8). The incidence of NBD in BD patients is approximately 9% (range, 3–30%) (9). The average age of NBD onset in children is around 12 years (10). Boys are more likely to develop NBD than adult men (2).

Here we report a pediatric case of NBD with intestinal lesions.

## Methods

### Clinical Examination

The medical history of the patient was provided by his parents, which included clinical manifestations, diagnosis, and treatment. Physical examinations, including fundus examination, and laboratory tests, including blood tests to assess the erythrocyte sedimentation rate and levels of C-reactive protein and inflammatory factors, were performed. Imaging analysis via magnetic resonance imaging and venography (MRI and MRV, respectively) were applied. Colonoscopy and histopathological examination were also performed.

## Results

### Case Presentation

The patient was a boy aged 11 years and 11 months and was admitted to our hospital due to abdominal pain and headache. His abdominal pain was localized to the right lower abdomen and was defined as paroxysmal and unbearable. He also had sporadic oral ulcers but no fever, rash, joint swelling, or joint pain. He underwent appendectomy due to appendicitis in the initial stage of abdominal pain. He also underwent surgery to remove his perianal abscess. He was the only child in his family, and his parents were healthy and had no similar medical history.

**Figure 1:**
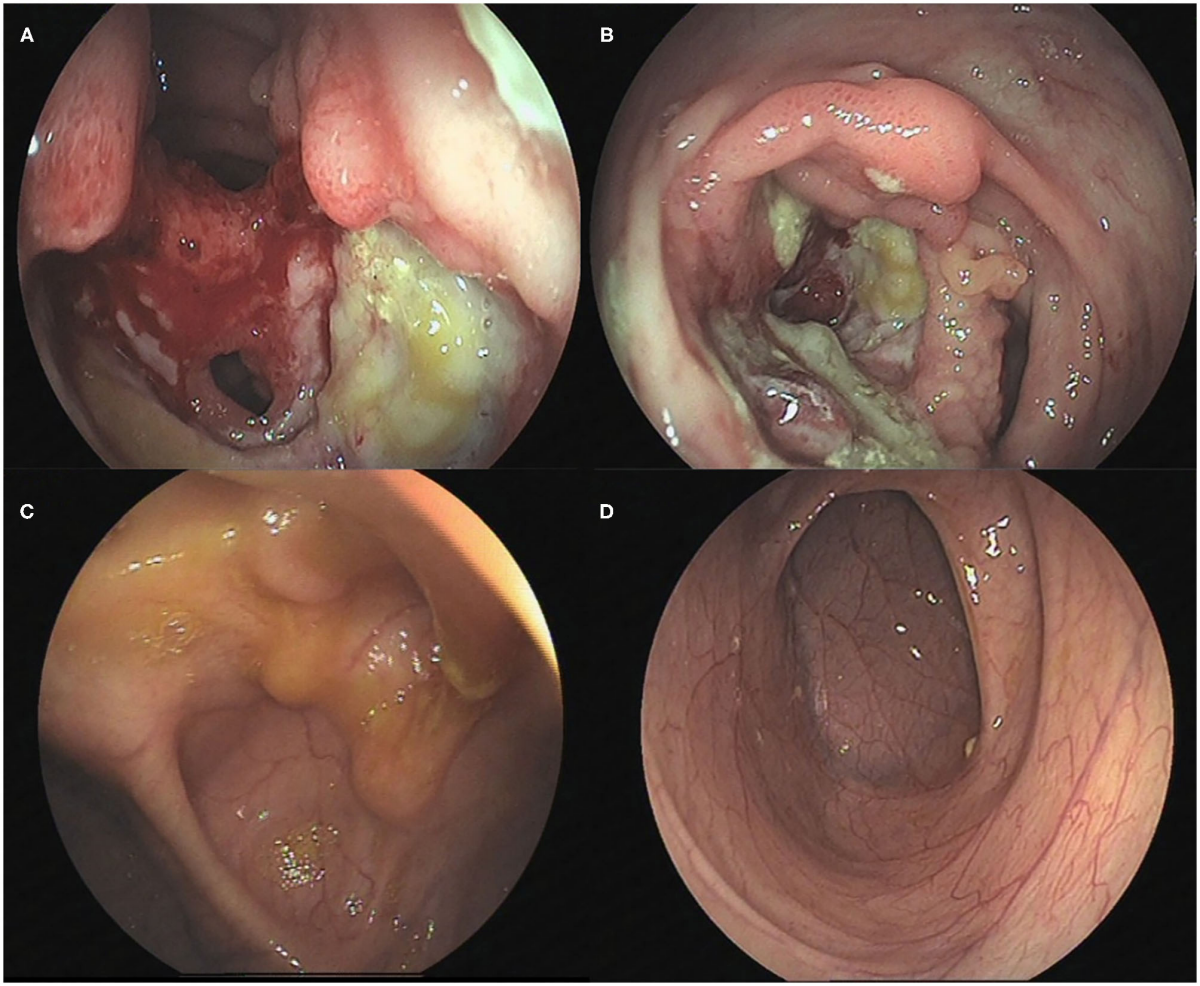
Colonoscopic changes in the patient. **(A)** The terminal ileum was covered with white moss with a large and flaky ulcer accompanied by erosion. **(B)** The mucosa was hyperemic and edematous, with a perforated intestinal wall with a diameter of ~1.5 cm. **(C)** Scattered inflammatory polypoid changes were observed in the mucosa around the ileocecal valve mouth, without erosion or ulcer, whereas the morphology of the ileocecal valve was deformed. **(D)** No abnormalities were observed in the mucosa of the terminal ileum.

**Figure 2:**
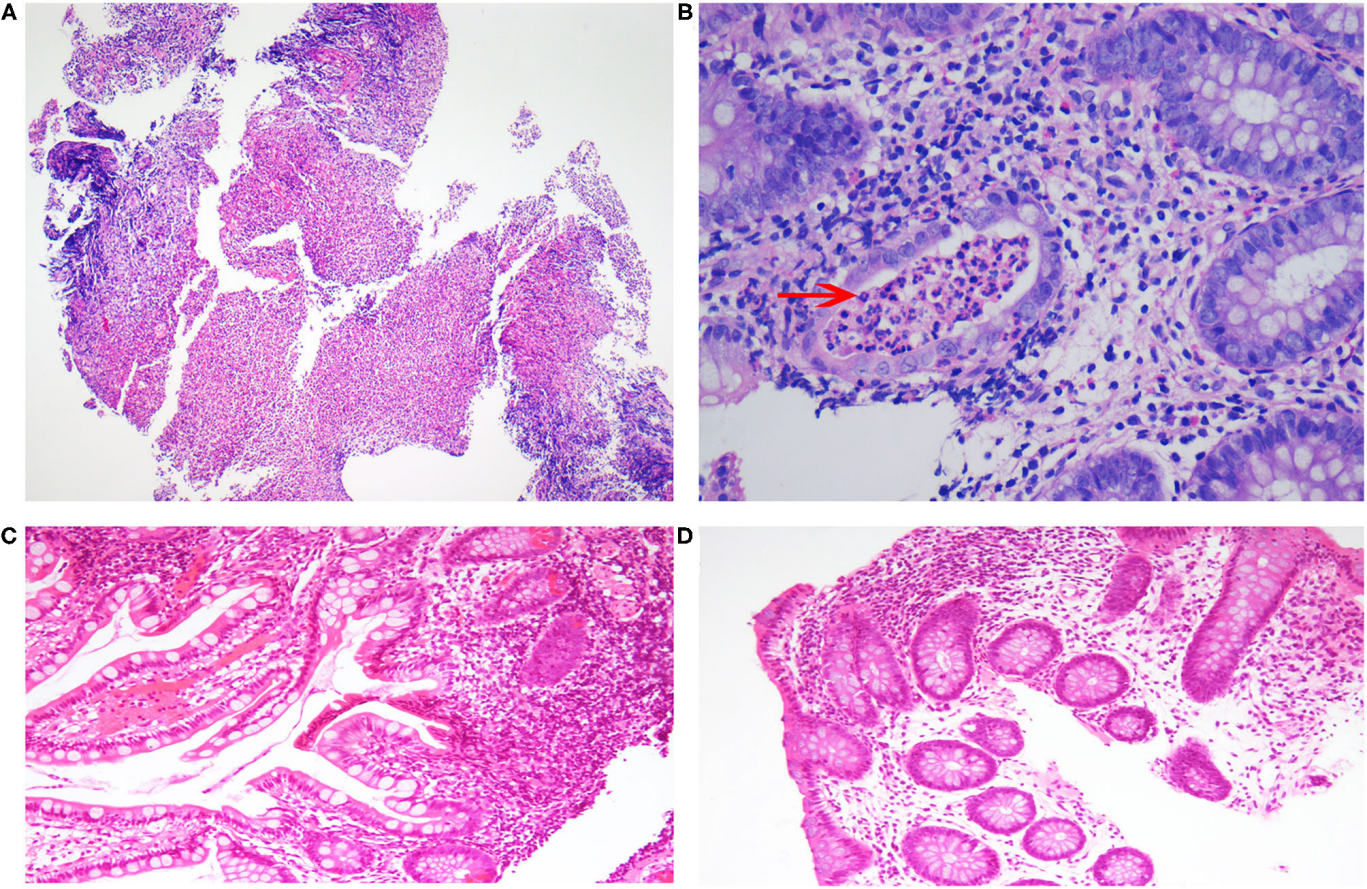
Histopathological changes in the patient. **(A)** The mucosal tissue of the ileocecal junction was denatured and necrotic, with ulcerative changes. **(B)** Neutrophils accumulated in the lumen of the gland with chronic inflammation, and a crypt abscess was seen (arrow). **(C)** A few neutrophils were seen infiltrating the mucosa of the transverse colon. **(D)** No obvious abnormality was observed in the intestinal crypt of the ileocecal mucosa.

Unfortunately, his abdominal pain and oral ulcers persisted. He was admitted to the hospital again and underwent electronic colonoscopy (Figures 1A,B) and pathological examination (Figures 2A,B), whereby Crohn's disease (CD) was diagnosed. His treatment course included mesalazine, infliximab, and briefly thalidomide. During the course of the treatment, he developed an anal fistula and continued to have abdominal pain.

One week before admission, he developed paroxysmal headache, without convulsions, blurry vision, tinnitus, weak limbs, vomiting, abdominal pain, diarrhea, fever, and other manifestations of NBD. The supplementary medical history revealed that he was diagnosed with uveitis in the right eye and received intraocular lens implantation in another hospital 3 years ago due to blurred and decreased vision in the right eye. Thus, the total duration of the disease was estimated to be 3 years.

**Figure 3:**
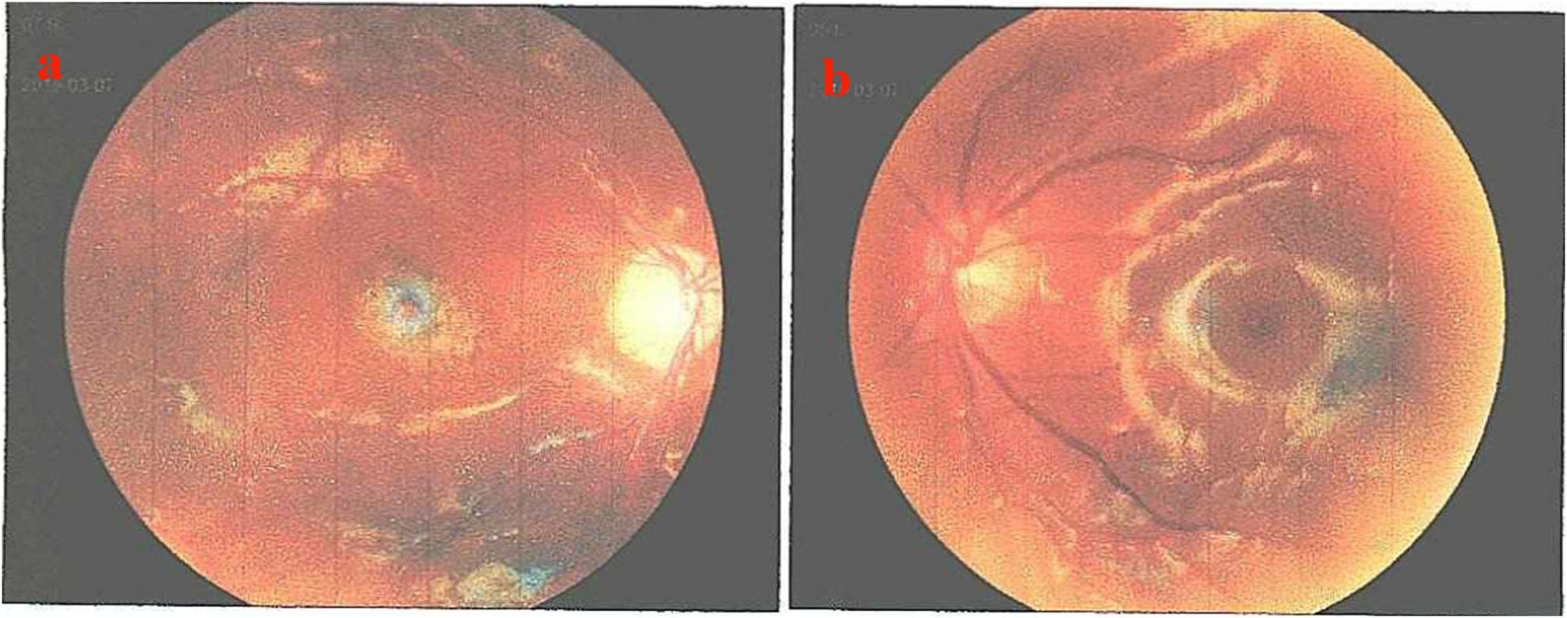
Ocular fundus changes in the patient. **(a)** No obvious abnormality was observed in the left fundus. **(b)** The cornea of the right eye was deformed, the anterior chamber was clear, the crystal membrane was white and cloudy, the intraocular lens was normal, the optic papilla was pale, and the macular area was yellow and disordered (C/D = 0.7).

Physical examination indicated that he had a splitting headache without any other positive signs, such as the Babinski sign, stiff-neck, and other neurological manifestations. The ocular fundus revealed that the cornea of the right eye was deformed. Nevertheless, the anterior chamber was still clear, the crystal membrane was white and cloudy, the intraocular lens was normal, the optic papilla was pale, and the macular area was yellow and disordered (C/D = 0.7) (Figure 3).

Laboratory tests revealed increased C-reactive–protein level, white–blood-cell number, and erythrocyte sedimentation rate. The count of Th1 and Th2 helper T-cell subsets indicated that IL-6, IL-2, IL-4, and IFN-γ levels were elevated (Table 1). Results from tests for hepatic, renal, and coagulation functions were normal. There was no abnormality in cerebrospinal fluid.

**Table 1:** Counts of Th1 and Th2 subsets of helper T cells.

**When**	**IL-2** **(pg/ml)**	**IL-4** **(pg/ml)**	**IL-6** **(pg/ml)**	**IL-10** **(pg/ml)**	**TNF-α** **(pg/ml)**	**TNF-γ** **(pg/ml)**
Before CD	61.31	5.44	192.99	0	5.05	5.62
Before NBD	10.48	6.65	21.34	1.8	0	5.55
After NBD	3.09	1.7	12.75	3.73	1.97	2.4

*Before CD: when the patient was admitted to our hospital half a year ago, before he was diagnosed with Crohn's disease. Before NBD: when the patient was admitted to our hospital at the onset of this study, after he was diagnosed with CD and before he was diagnosed with NBD. After NBD: ~0.5 years after he was diagnosed with NBD. IL, interleukin; TNF, tumor necrosis factor*.

**Figure 4:**
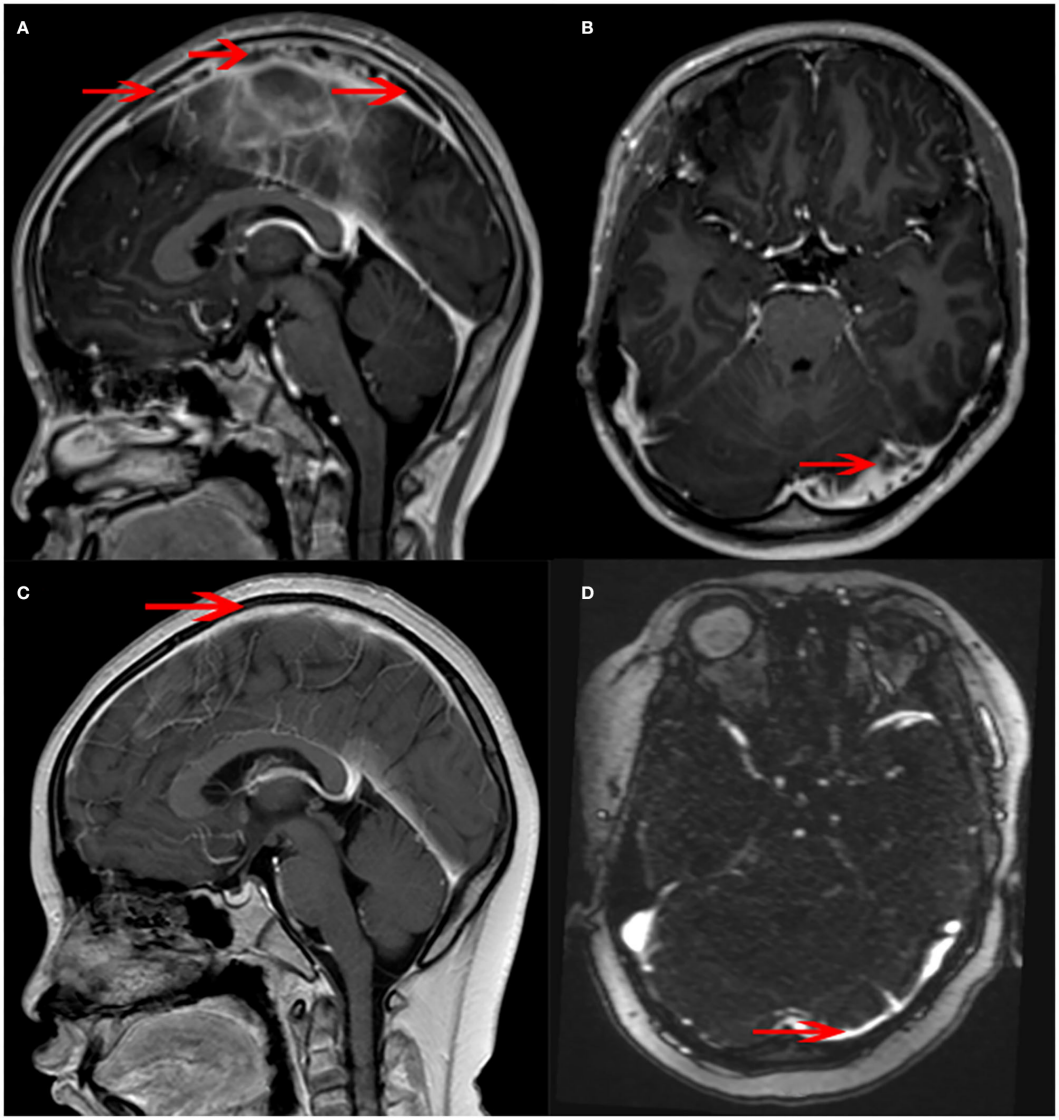
Magnetic resonance venography (MRV) changes in the patient. **(A)** There were many banded filling defects in the superior sagittal sinus (arrow). **(B)** A few filling defects were seen in the left transverse sinus (arrow). **(C)** The superior sagittal sinus was slightly narrowed, and the margin was rough (arrow). No obvious intracranial venous thrombosis was observed. **(D)** The left transverse sinus was slightly thin (arrow).

The MRI and MRV of the brain showed many filling defects in the superior sagittal, left sigmoid, and bilateral transverse sinuses, later diagnosed as cerebral venous sinus thrombosis (CVST). No abnormal signal shadow was observed in the brain parenchyma, ventricle, brainstem, or cerebellum (Figures 4A,B).

### Treatment and Prognosis

The findings above resulted in his diagnosis of NBD. He was treated with an anticoagulation agent (nadroparin calcium), low-dose corticosteroids, and an immunosuppressant (cyclophosphamide). Fortunately, after 6 months, his clinical symptoms and signs disappeared. The levels of inflammatory cytokines significantly decreased (Table 1). The electronic colonoscopy and pathological examination indicated that his bowel was recovering well (Figures 1C,D, 2C,D). MRI of the head showed that there was no definite intracranial venous sinus thrombosis, and the superior sagittal sinus was slightly narrow with a rough margin, while the sigmoid sinus, transverse sinus, and left internal jugular vein were slightly thin (Figures 4C,D). These results from brain imaging indicated neurological improvement. For the next year, he was on low-dose oral glucocorticoids, rivaroxaban, and linezolid, and completed four cyclophosphamide shock treatments, without any adverse effects, except for vision problems.

## Discussion and Conclusion

NBD in children is different from that observed in adults. It is reported in 3.6–59.6% of pediatric BD cases, and the incidence of NBD-related cerebrovascular diseases is higher in children than in adults (11, 12). Metreau-Vaste et al. (6) have found that the most common neurological lesion is CVST, whereas meningoencephalitis and peripheral neuritis are rare. In their study, two of the 12 children (17%) had abdominal pain with severe colonic ulcers. Our case also presented with abdominal pain and intestinal mucosal ulcers. Previous studies have reported that such cases are relatively rare.

It has been reported that the most common initial neurological symptoms are headache and epileptic seizure. Cranial neuropathy and hemiplegia may also occur in NBD, but the most common type of neurological involvement is CVST (2). Through MRI and MRV, CVST can be detected most prominently in transverse and superior sagittal sinuses. In a few children, the brain stem and spinal cord may be involved (2). The CVST in our case mainly occurred in the transverse and superior sagittal sinuses (Figures 4A,B).

The uveitis in the right eye, appendicitis, and perianal abscess in this case resulted from NBD. However, these symptoms may be easily ignored in clinical practice. This patient has undergone intraocular lens implantation, appendectomy, and perianal abscess removal. Abdominal pain was the main symptom in the patient. He was misdiagnosed with CD based on the colonoscopy findings.

As intestinal BD and CD share many common characteristics, including genetic factors, clinical manifestations, and courses of pathogenesis and treatment, it is difficult to distinguish between the two diseases. Some experts believe that the two diseases are simply different clinical manifestations of the same disease, but others think BD and CD are completely different diseases (12). The major differences between these two diseases are the clinical manifestations; BD results in oral and genital ulcers and involves the nervous system, occlusive vascular disease, and thrombosis, whereas patients with CD generally have anal complications, such as stenosis, abscess formation, and fistula, which are rare in BD (13). However, the perianal abscess in our case might have been easily misdiagnosed as CD. Nevertheless, colonoscopy can reveal the differences between the two diseases. BD is localized in the terminal ileum or ileocecal region and usually has single or several, discrete, large, and round or oval ulcers. The classical endoscopic appearance of CD involves discontinuous mucosal inflammation, longitudinal ulcer, and pebbly appearance of the normal surrounding mucosa. In our patient, the presentation was concentrated in the ileocecal region (Figures 1A,B), more in line with BD than CD. The pathological manifestations of CD are cryptitis and non-caseous granuloma, whereas those of BD are vasculitis, mucosal inflammation, with/without ulcerative (12, 14). Pathologically, the patient had changes in the crypts (Figures 2A,B), similar to those seen in CD. Moreover, CD has been reported with extensive thrombosis in the limbs and CVST (15–19). A study has reported that the thrombosis prevalence among patients with CD is 5.7% (20). In addition, BD is frequently misdiagnosed as CD in adults (21). Overall, CD has many features that overlap with BD, and as our case shows, distinguishing between these two conditions can be challenging.

Therefore, the patient was initially diagnosed with CD because of its clinical presentation. For children, CD can be distinguished from BD when the disease is accompanied by oral ulcers, and the colonoscopic lesions are limited to the ileocecal area (round and oval ulcers). Pathological and parenteral manifestations should be considered, and the diagnosis should be made after reviewing the history again. Furthermore, the MRI of the head or MRV may be considered. If CVST is detected, NBD can be diagnosed.

The therapeutic goal of NBD is to inhibit the progression and recurrence of inflammation to prevent irreversible organ damage. The first-line drugs against NBD are high-dose glucocorticoids, cyclophosphamide, and azathioprine, whereas refractory NBD can be treated with second-line drugs, such as anti-tumor necrosis factor (TNF)-α or tocilizumab (14). However, additional reliable evidence is still needed to prove the efficacy and safety of biologics in the treatment of pediatric NBD.

In conclusion, NBD is prone to misdiagnosis and missed diagnosis and should be diagnosed based on clinical manifestations and results from colonoscopy, pathological examination, and MRI or MRV.

## Data Availability Statement

The original contributions presented in the study are included in the article/supplementary material, further inquiries can be directed to the corresponding author.

## Ethics Statement

The studies involving human participants were reviewed and approved by Ethics Committee of Shenzhen Children's Hospital. Written informed consent to participate in this study was provided by the participants' legal guardian/next of kin. Written informed consent was obtained from the individual(s), and minor(s)' legal guardian/next of kin, for the publication of any potentially identifiable images or data included in this article.

## Author Contributions

QZ and ZW: conceptualization and writing original draft. YL and JZ: data collection. QZ and SZ: formal analysis. ZW: funding acquisition. QZ: investigation. QZ, YL, JZ, SZ, and ZW: writing-review and editing. All authors have read and approved the manuscript.

## Funding

This work was supported by Shenzhen Fund for Guangdong Provincial High level Clinical Key Specialties (no. SZGSP012).

## Conflict of Interest

The authors declare that the research was conducted in the absence of any commercial or financial relationships that could be construed as a potential conflict of interest.

## Publisher's Note

All claims expressed in this article are solely those of the authors and do not necessarily represent those of their affiliated organizations, or those of the publisher, the editors and the reviewers. Any product that may be evaluated in this article, or claim that may be made by its manufacturer, is not guaranteed or endorsed by the publisher.

## Abbreviations

BD: Behcet's disease
NBD: Neuro-Behcet's disease
CD: Crohn disease
CVST: Cerebral Venous Sinus Thrombosis
MRI: magnetic resonance imaging
MRV: magnetic resonance venography
HLA-B51: human leukocyte antigen-B51
TNF: tumor necrosis factor.
